# Inulin Amphiphilic Copolymer-Based Drug Delivery: Unraveling the Structural Features of Graft Constructs

**DOI:** 10.3390/pharmaceutics16080971

**Published:** 2024-07-23

**Authors:** Carla Sardo, Giulia Auriemma, Carmela Mazzacano, Claudia Conte, Virgilio Piccolo, Tania Ciaglia, Marta Denel-Bobrowska, Agnieszka B. Olejniczak, Donatella Fiore, Maria Chiara Proto, Patrizia Gazzerro, Rita Patrizia Aquino

**Affiliations:** 1 Department of Pharmacy, University of Salerno, Via Giovanni Paolo II 132, 84084 Fisciano, Italy; gauriemma@unisa.it (G.A.); carmela.mazzacano@unina.it (C.M.); tciaglia@unisa.it (T.C.); dfiore@unisa.it (D.F.); maproto@unisa.it (M.C.P.); pgazzerro@unisa.it (P.G.); aquinorp@unisa.it (R.P.A.); 2 Department of Pharmacy, University of Naples Federico II, Via Domenico Montesano 49, 80131 Napoli, Italy; claudia.conte@unina.it (C.C.);; 3 Institute of Medical Biology, Polish Academy of Sciences, Lodowa 106, 93-232 Lodz, Poland; mdenel-bobrowska@cbm.pan.pl (M.D.-B.); aolejniczak@cbm.pan.pl (A.B.O.)

**Keywords:** PEG alternative, inulin, PLA, sorafenib tosylate, crew-cut nanoparticles, FALT

## Abstract

In this study, the structural attributes of nanoparticles obtained by a renewable and non-immunogenic “inulinated” analog of the “pegylated” PLA (PEG-PLA) were examined, together with the potential of these novel nanocarriers in delivering poorly water-soluble drugs. Characterization of INU-PLA assemblies, encompassing critical aggregation concentration (CAC), NMR, DLS, LDE, and SEM analyses, was conducted to elucidate the core/shell architecture of the carriers and in vitro cyto- and hemo-compatibility were assayed. The entrapment and in vitro delivery of sorafenib tosylate (*ST*) were also studied. INU-PLA copolymers exhibit distinctive features: (1) Crew-cut aggregates are formed with coronas of 2–4 nm; (2) a threshold surface density of 1 INU/nm^2^ triggers a configuration change; (3) INU surface density influences PLA core dynamics, with hydrophilic segment stretching affecting PLA distribution towards the interface. INU-PLA_2_ *NPs* demonstrated an outstanding loading of *ST* and excellent biological profile, with effective internalization and *ST* delivery to HepG2 cells, yielding a comparable IC_50_.

## 1. Introduction

Biocompatible, amphiphilic block copolymers, especially those employing polyethylene glycol (PEG) as the hydrophilic block, have been extensively investigated for their potential in constructing core/shell structures [1,2]. However, the clinical application of PEGylated nanosystems has faced challenges, such as accelerated blood clearance (ABC) in a subpopulation of individuals who develop antibodies against PEG [3]. Therefore, it is crucial to investigate alternative materials that can overcome this restriction while still preserving the benefits linked with PEGylation [4,5,6].

Potential alternatives to PEG have been assessed among various synthetic and natural polymers, including poly(*N*-vinylpyrrolidone), poly(2-oxazolines), poly(glycerols), hyaluronic acid, and polyaminoacids [7,8,9,10]. A thorough comparison reveals that these potential substitutes do not yet meet the rigorous standards established by PEG, often regarded as the gold standard. Key deficiencies frequently pertain to colloidal stability, biocompatibility, immune system interaction, degradation, excretion, and bioaccumulation.

Amphiphiles, such as PLGA-g-PVP [11], showed promising results but those preliminary data need to be substantiated by further specifically addressed research, such as systematic and standardized investigations into their chemico-physical properties. This could provide new perspectives for comparison and facilitate the development of predictive algorithms based on the evaluation of structural features, assembly dynamics, and supramolecular interactions.

Inulin (INU), a naturally occurring polysaccharide abundant in plants, has garnered significant attention as a versatile biomaterial for the development of nanomedicines [12,13,14]. Several conjugates were designed to have a hydrophilic inulin backbone as the external shell and hydrophobic moieties as the internal core. Those derivatives were found to be an effective and stable delivery strategy for overcoming the poor water-solubility of many drugs. Once the approach is validated and a systematic evaluation is conducted on how the chemical modification of inulin with hydrophobic moieties enhances its amphiphilicity and suitability for constructing stable and efficient drug delivery platforms, it is necessary to compare the half-lives of encapsulated versus free forms of a drug. This provides crucial insights into the overall therapeutic effectiveness. In some reports, INU amphiphiles-based nanosystems significantly prolong the presence of drugs in circulation, enhancing their potential therapeutic impact [15,16].

Recently, it has been explored as a renewable, hydrophilic building block to address challenges associated with PEGylation in nanomedicine. PLA was grafted onto INU to generate an amphiphilic, biodegradable copolymer capable of self-assembling into core/shell *NPs*. The proposed material (INU-PLA) represents an “inulinated” graft analog of the “PEGylated” di-block PEG-PLA [17,18]. Although the initial study demonstrated that INU-PLA can self-assemble into nanostructures smaller than 200 nm in size and is capable of loading a moderately water-soluble drug, such as doxorubicin hydrochloride, with an encapsulation efficiency comparable to that of PEG-PLA analogs, little was still known about its dispersion dynamics in terms of critical aggregation concentration, core/shell structure, the thickness of the inulin layer, and its conformation. These pieces of information are essential, considering that these properties are typically correlated with colloidal stability, dilution resistance, stealth properties, circulation time, and the formation of a hard protein corona on the nanosystem surface following intravenous administration. Furthermore, while extensive research has been conducted on *NPs* derived from block amphiphilic copolymers such as PEG-PLA, exploration of graft constructs featuring hydrophobic chains pendant from a hydrophilic backbone remains relatively limited. One rare exemplificative case is poly(*ε*-caprolactone) functionalized hyperbranched poly(glycerol) for which a “unimolecular micelle-like” behavior has been demonstrated [19]. Existing literature provides only a few examples, and even in these cases, obtaining comprehensive information regarding their assembly behavior and supramolecular architecture remains challenging.

Therefore, this study aims to (1) investigate the structural features of graft amphiphiles and (2) provide a comprehensive characterization of INU-PLA assemblies, including critical aggregation concentration (CAC) and water solubility, through analyses of NMR, DLS, LDE, and SEM to elucidate the carrier’s core/shell structure. Additionally, in vitro biological characterization of INU-PLA is reported for the first time. Cytocompatibility and hemocompatibility studies were conducted for INU-PLA, and to demonstrate the potential clinical relevance of these novel nanocarriers in the delivery of drugs with negligible water solubility, the incorporation of *ST* allowed evaluation of encapsulation efficiency and release, cellular uptake, and in vitro drug efficacy.

## 2. Materials and Methods

### 2.1. Materials and Equipment

INU from dahlia tubers (Mw ≈ 5000 Da), sorafenib tosylate (*ST*), triethylamine (TEA), carbonyl diimidazole (CDI), diethyl ether (EtOEt), acetone, dimethylformamide, dimethyl sulfoxide (DMSO), and pyrene were purchased from Merck (Darmstadt, Germany). Poly(D,L-lactide) acid endcap (P-D,L-LA_1K_, Mn: 1000–5000) and poly(D,L-lactide) acid endcap (P-D,L-LA_5K_, Mn: 5000–10,000) were obtained from PolySciTech (Division of Akina, West Lafayette, IN, USA). The dispersities of the PLA utilized and the respective GPC chromatograms are reported in Supplementary Materials (SM) Figure S1. INU was oven-desiccated at 70 °C for 15 to 20 h and cooled under a vacuum over P_2_O_5_ prior to being used. Solvents were not purified before being used. Dialyses tubing (regenerated cellulose) was from Spectrapor (Repligen, Waltham, MA, USA). NMR spectra were obtained from samples dissolved in DMSO-d_6_ on a spectrometer operating at 400 MHz (Bruker Avance III, Rheinstetten, Germany). FT-IR spectra were acquired on an ATR single reflection sampling module by placing the solid samples, using a Frontier FT-IR spectrometer (Perkin Helmer, Waltham, MA, USA).

### 2.2. INU-PLA and INU-FITC-PLA Synthesis

Derivatization of INU with PLA to obtain the INU-PLA graft copolymers was achieved according to the procedure reported by Sardo et al. [20]. ^1^H NMR (400 MHz, DMSO-d6) and FT-IR (ATR) were as previously reported. The same procedure was applied to obtain a fluorescent derivative of INU-PLA_2_ functionalized with fluorescein isothiocyanate starting from INU-FITC, purchased from Merck.

### 2.3. Copolymers Self-Assembly

CAC of the copolymers was determined by a fluorescence spectroscopy method [21]. In brief, copolymers were stirred for 24 h in water at 60 °C. The dispersions were cooled and filtered to remove undissolved polymer. To determine the polymer concentration, 1 mL of the filtrate was withdrawn and freeze-dried, and the weight after freeze-drying was taken as the solubility expressed in mg/mL. A solution containing pyrene at a concentration of 6.0 × 10^−5^ M in acetone was initially made. Subsequently, portions of 20 μL from this solution were dispensed into vials and allowed to stand for the acetone to volatilize (in the dark). Aqueous polymer solutions at different concentrations ranging from 8 × 10^−5^ to 1000 μg/mL were added to the residue, and samples were heated to 65 °C for 1 h, cooled to room temperature, and analyzed at 25 °C on a Shimadzu RF-6000 spectrofluorometer, Shimadzu, Japan. Excitation spectra were registered at λ_em_ = 390 nm, and the ratio I334/I331 was plotted against polymer concentration. CAC was extrapolated from the plots of pyrene I334/I331 ratio versus the logarithm of the copolymer concentration. The plot was described by a sigmoid of the type:

(1) $$y=min+\frac{min-max}{{1+e}^{(x-half)/Slope}},$$

where the variable *y* corresponds to the pyrene I334/I331 ratio value; the independent variable (*x*) is the concentration of copolymer; *min* and *max* are the lower and upper limits of the sigmoid, respectively; *half* is the center of the sigmoid; and *slope* is directly related to the independent variable range where the abrupt change of the dependent variable occurs. Given *min*, *max*, *half*, and *slope* as fitting parameters of the experimental data, the CAC was calculated as follows:

CAC = *half* + 2·*Slope*, (2)

### 2.4. NPs Production

#### 2.4.1. Pre-Assembling/Dialysis Method

Thirty milligrams of INU-PLA were dissolved in 0.6 mL of DMF containing 6 mg of *ST*. The solution was sonicated in an ultrasonic bath, and 10 mL of double distilled water was gradually added. Afterwards, the mixture was tip-sonicated for 2 min (50 °C, 25% amplitude) with a Q700 Fisher Scientific sonicator. The dispersion was cooled to room temperature under vigorous stirring, and DMF was removed by dialysis against double distilled water (25 KDa MWCO, 4 h, 2 changes of 2 L each). The dispersion was then filtered through a 0.45 µm Regenerated Cellulose (RC) syringe filter. The filtrate was flash-frozen and subsequently freeze-dried to obtain a white solid.

#### 2.4.2. Pre-Assembling/Film Rehydration Method

Ten milligrams of INU-PLA were dissolved in 0.2 mL of DMSO containing 2 mg of *ST*. The solution was sonicated in an ultrasonic bath, and 3 mL of water was gradually added. The mixture was sonicated for 5 min. A further aliquot of water (7 mL) was then gradually added under continuous sonication for 10 min. The resulting mixture was flash-frozen and freeze-dried overnight at 0.1 mbar. The obtained film was redispersed in water, as reported above, and filtered through a 0.45 µm RC syringe filter. The filtrate was flash-frozen and then freeze-dried to obtain a white solid.

### 2.5. Determination of the Drug Loading (DL%)

A precisely weighted amount of freeze-dried drug-loaded *NPs* was dissolved in DMSO (final concentration around 0.1 mg/mL) and the absorbance read at 270 nm using a spectrophotometer Evolution 201 (Thermo Scientific, Waltham, MA, USA) in a quartz cuvette with an optical path of 1 cm. The drug content was extrapolated from a calibration curve obtained by measuring *ST* solution under the same conditions with a known concentration in the range of 1 to 20 μg/mL. Supplementary Materials Figure S2 reports a representative calibration curve. *DL*% was calculated as follows:

(3) $$DL\%=\frac{mass\;of\;ST\;in\;NPs}{mass\;of\;NPs\;sample}\times 100,$$

### 2.6. Size, Distribution, ζ Potential

DLS measurements were performed at 25 °C with a Malvern Zetasizer Nano ZS instrument. The intensity-average hydrodynamic diameter (nm) and polydispersity index (PDI) were obtained by cumulative correlation function analysis. z potential measurements were performed by aqueous electrophoretic light scattering measurements, under the same conditions, using the same instrument. The z potential values (mV) were calculated from the electrophoretic mobility using the Smoluchowsky relationship. Samples were dispersed in double-distilled water at 1 mg/mL and sonicated for 60 s before measurement.

### 2.7. Ultra-Hight Resolution Scanning Electron Microscopy (UHR-SEM)

The morphology of representative formulations was studied by SEM (Tescan Solaris, Tescan Orsay Holding, Brno, Czech Republic). Analysis was conducted at 20 KeV. *NPs*, after preparation and in water dispersion, were dropped on a carbon-coated aluminum stub and dried from water under a gentle nitrogen stream overnight. Before capturing images, the samples underwent a gold sputter-coating process.

### 2.8. In Vitro Drug Release Study

An experiment was conducted to study the release of substance *ST* in vitro, utilizing the dialysis technique under sink conditions. This was carried out in a phosphate buffer solution (PBS) with a pH of 7.4, mimicking physiological fluid. An amount of drug-loaded INU-PLA_2_ *NPs*, corresponding to 296 µg of *ST*, was dispersed in 1 mL of PBS. The dispersion was immediately transferred into a cellulose ester dialysis tubing (Spectra-Por^®^ RC, MWCO 25 kDa) and immersed into 50 mL of PBS containing 10% (*v*/*v*) of Tween 80. The system was incubated at 37 °C under continuous stirring (100 rpm) in an orbital shaker. One-milliliter aliquots of the external medium were withdrawn from the acceptor at fixed time intervals and replaced with equal volumes of fresh medium. Samples were freeze-dried. The amount of *ST* was detected by redispersion of dry samples in DMSO and measuring the absorbance at 270 nm. A calibration was made by serial dilution of a *ST* solution in DMSO. For comparison, the diffusion of *ST* from a saturated solution in PBS containing 1% Tween 80 was followed across the same membrane for 24 h.

### 2.9. Fixed Aqueous Layer Thickness (FALT) Determination

FALT measurements relied on an approximation derived from the Gouy–Chapman theory, employing the method of monitoring the impact of ionic strength on the particle surface [22]. Various concentrations of NaCl stock solutions were mixed with an *NP* dispersion in double distilled water (0.5 mg/mL). Subsequently, the ζ potential of each sample was determined. The relationship between the natural logarithm of the absolute value of ζ potential and the square root of the NaCl concentration was analyzed to determine the thickness of the hydrophilic shell in nanometers (representative plots are reported in Figure S3 in Supplementary Materials).

### 2.10. Surface INU Density Determination

The surface INU density on *NPs* was determined by ^1^H NMR [23,24] using Bruker instrument at 400 MHz, Rheinstetten, Germany. INU-PLA_1–4_ *NPs* were prepared directly by solvent displacement, diluting a copolymer solution in DMSO-d_6_ (10 mg in 20 µL) with D_2_O with 0.2 wt% sodium acetate as an internal standard. A precisely weighted amount of INU was dissolved in the same solvent and serially diluted to generate a calibration curve (representative spectra and a calibration curve are reported in Figure S4 in Supplementary Materials). The surface INU density (Γ) was calculated as the number of INU chains per 100 nm^2^ nanoparticle (*NP*) core surface as follows:

(4) $$[\Gamma ]=\frac{M\times 6.02\times 10^{23}}{\frac{\frac{W}{d}}{\frac{4}{3}\pi \left(\frac{D}{2}\right)3}}/4\pi {\left(\frac{D}{2}\right)}^{2}\times 100,$$

where *d* is the core density (1.21 g/cm^3^ for PLA), *W* is the total mass of the sample tested (g), and *D* is the particle core diameter calculated as the average diameter obtained by DLS (Z-average, nm) minus the shell thickness obtained by FALT.

### 2.11. Hemolysis and Erythrocyte Aggregation Evaluation

Freshly collected human blood from a healthy donor was centrifuged at 2200 rpm for 10 min (Thermo Scientific Heraeus Labofuge 200 Centrifuge, Waltham, MA, USA) to isolate red blood cells (RBCs). The RBC pellet was dispersed gently in isotonic 10 mM Dulbecco modified phosphate buffer saline (DPBS) solution (pH 7.4) and isolated again by centrifugation. About 0.35 mL of the finally diluted RBC suspension (4% *v*/*v* in DPBS) was added to 0.35 mL of a NP dispersion to obtain a concentration ranging from 25 to 1000 μg/mL. After incubation at 37 °C for 1 h, the samples were centrifuged at 2200 rpm on a Minispin^®^ centrifuge (Eppendorf, Hamburg, Germany) for 10 min to remove non-lysed RBCs. The supernatant was examined to measure the release of hemoglobin using spectrophotometric determination at a wavelength of 540 nm. As the positive control (100% hemolysis), 0.35 mL of a sodium dodecyl sulfate solution [25] (final concentration 180 μM) was added to 0.35 mL of the finally diluted RBC suspension and treated as reported above. As the negative control (0% hemolysis), 0.35 mL of DPBS was added to 0.35 mL of the finally diluted RBC suspension and treated the same way. The hemolysis was determined by the following equation:

(5) $$hemolysis\;\%=\frac{Abs-Abs0}{Abs100-Abs0}\times 100,$$

where *Abs*100 and *Abs*0 are the absorbances of the solution at 100 and 0% hemolysis, respectively. To evaluate morphological changes and aggregation of RBCs, samples were examined by microscopy observation (Microscope B-190TBPL, Optika, Ponteranica, Italy; objective Optika N-PLAN 40×) after deposition on glass coverslips. Images were acquired.

### 2.12. Cell Culture

The human hepatocyte carcinoma cell line (HepG2) was obtained from the American Type Culture Collection (ATCC). Cells were cultured in Eagle’s Minimum Essential Medium (EMEM), supplemented with 10% (*v*/*v*) Fetal Bovine Serum (FBS), 2 mM L-glutamine, and 100 U/mL penicillin–streptomycin (all reagents were from Sigma-Aldrich, Darmstadt, Germany), at 37 °C, 5% CO_2_. The cell line was routinely screened and confirmed to be negative for mycoplasma contamination.

### 2.13. Cell Viability by MTT Assay

HepG2 cells were plated in 96-well microtiter plates (5 × 10^3^ cells/well). After 24 h, cells were incubated for 72 h with fresh medium containing *ST*, dissolved in DMSO at the range concentration of 0.6–10 μM; known amounts of *ST*-loaded *NPs* corresponding to the entrapped *ST* concentration in the 0.6–10 μM range and finally empty *NPs* by using the same concentrations used for *ST*-loaded *NPs*. Samples were suspended in twice-distilled water in sterile conditions, sonicated for 20 min, and diluted with EMEM. After incubation with drugs, the cells were treated with 3-(4,5-dimethylthiazol-2-yl)-2,5-diphenyltetrazolium bromide dye solution (MTT, Sigma-Aldrich, Darmstadt, Germany) (25 μL, 5 mg/mL) for 2 h, lysed by overnight incubation at 37 °C with 100 μL of SDS solution (13.5% *wt*/*v* in DMF/distilled water 45:55) (100 μL). Subsequently, the optical density at 550 nm, along with a reference wavelength of 670 nm, was assessed using a microplate spectrophotometer (Varioskan Lux; Thermo Fisher Scientific, Waltham, MA, USA). Relative cell viability (percentage) was expressed based on three independent experiments. Medium-only treated cells under the same conditions were used as a negative control. In each experiment, the IC_50_ value was determined as the concentration at which 50% inhibition of cell viability occurs.

### 2.14. Cellular Uptake by Flow Cytometry (FACS) Analysis of INU-PLA_2_ FITC NPs

For the FACS analysis, HepG2 cells were seeded in 6 well plates (2 × 10^5^ cells/well) in an adherent condition. After overnight adherence, cells were exposed to 20, 50, 100, and 250 µg of INU-PLA_2_ FITC *NPs* for 2 h or 4 h, and untreated cells were used as negative control. At the end of each time point, HepG2 cells were collected as single cells using trypsin-EDTA, centrifuged at 1200× *g* rpm for 5 min, and washed with 1× PBS (Phosphate-buffered Saline) three times. The amount of FITC-containing cells from the total cellular suspension was analyzed using a Becton Dickinson FACScan flow cytometer, Mountain View, CA, USA. Therefore 10,000 events were collected and corrected for debris and aggregate populations. Data were analyzed with FlowJo software 10.1 (BDIS) and are expressed as mean ± SD of three independent FACS experiments in triplicate.

### 2.15. Confocal Microscopy

HepG2 cells were grown in adhesion on slides in 24 well plates. After treatment for 4 h with 100 µg or 250 µg of FITC-*NPs*, cells were washed with 1× PBS and fixed in paraformaldehyde (PFA, 3.7% *v*/*v* in PBS) for 10 min. At the end, the cells were washed and permeabilized in Tryton X-100 (0.1% *v*/*v* in PBS) for 10 min. Then, cells were blocked with 4% Bovine Serum Albumin (BSA) for 1 h at RT and incubated with anti-EpCAM (epithelial cell adhesion molecule) primary antibody (1:500, Abcam, Waltham, MA, USA) for 1 h at RT. Immunofluorescence staining was obtained by incubating for 1 h (RT, in the dark) with Alexa Fluor 555 dye (tetramethylrhodamine conjugated) secondary antibody (1:500, Thermo Fisher Scientific). Nuclei were stained with 4′,6-diamidino-2-phenylindole (DAPI) dye (Thermo Fisher Scientific). The slides were mounted using a mowiol mounting medium and vertically scanned from the bottom by using a 63× (1.40 NA) Plan-Apochromat oil-immersion objective through the Leica SP8 confocal microscope (Leica Microsystems CMS Gmbh, Wetzlar, Germany). Quantifications of FITC-INU-PLA_2_ fluorescence intensity were performed using ImageJ software 8 (National Institutes of Health, Bethesda, MD, USA), calculating integrated densities per area from the FITC channel and subtracting background readings. The obtained mean value was used to compare experimental groups.

## 3. Results and Discussion

### 3.1. Copolymer Aggregation Threshold and Solubility

**Scheme 1:**
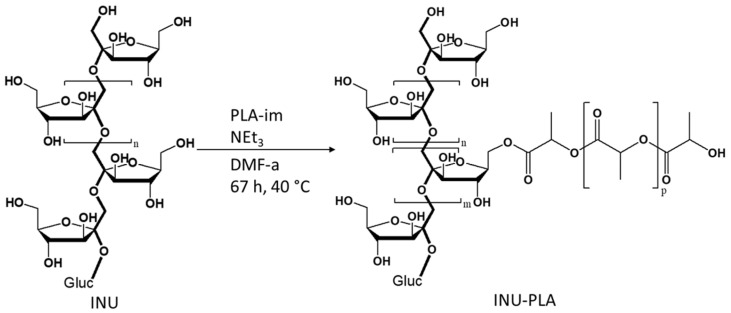
Reaction to obtain INU-PLA_1-4_ copolymers.

INU-PLA_1–4_ is a family of amphiphilic derivatives proposed as PEG-PLA alternatives and synthesized as reported previously [20]. Their chemical structure as well as the synthetic route is summarized in Scheme 1.

The various INU-PLAs differ for the Mn of the grafted poly-D,L-lactide (PLA) and for the amount of chains grafted per 100 fructose repeating units (DD_mol_% PLA). The molecular characteristics of INU-PLA_1–4_ copolymers in this study are reported in Table 1.

**Figure 1:**
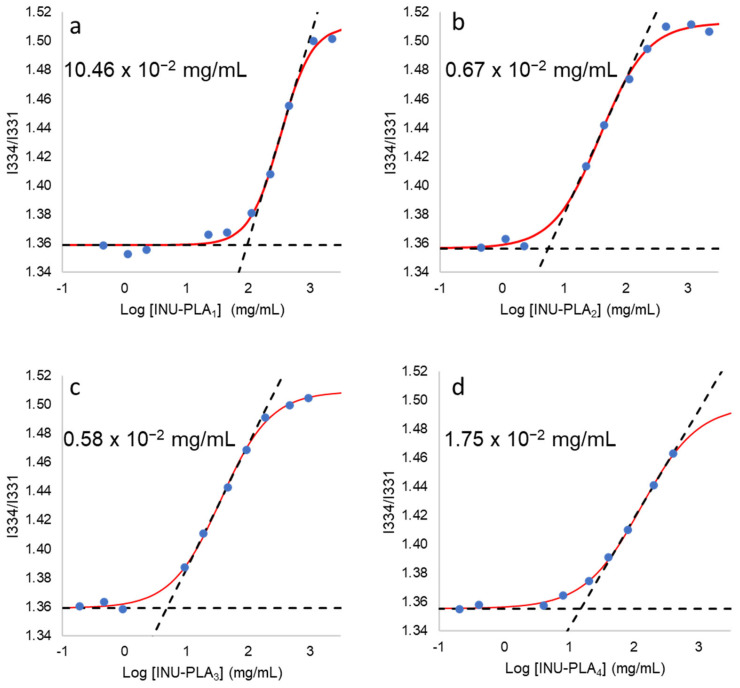
CAC determination for INU-PLA_1-4_ copolymers by pyrene method. Plots of the I334/I331 versus the logarithm of the copolymer concentration (mg/mL) for (**a**) INU-PLA_1_, (**b**) INU-PLA_2_, (**c**) INU-PLA_3_, and (**d**) INU-PLA_4_.

To assess the stability of a polymeric self-assembling system when subjected to the dilution effects inherent to i.v. injection processes, it is imperative to quantify the *NPs*’ CAC. The CAC of INU-PLA_1–4_ was measured employing pyrene as a fluorescent probe (Figure 1). The study revealed the lowest CAC for INU-PLA_2_ and INU-PLA_3_, which exhibited values ranging from 3.5 × 10^−^⁴ to 5 × 10^−^⁴ mM, as presented in Table 1. These observed CAC values are lower compared to those documented in the literature for INU-based amphiphilic polymers, typically possessing shorter hydrophobic segments such as squalene [26], tocopherol [27], or lauryl chains [18]. The difference in CAC values could be attributed to the presence of an elongated hydrophobic block, facilitating more robust hydrophobic interactions between copolymer chains within the core, thereby promoting their aggregation and the formation of micelles or other self-assembled structures in solution.

It is vital to underscore the pivotal role played by the equilibrium between the hydrophilic and hydrophobic components of the copolymer. When the hydrophobic block becomes overly represented relative to the hydrophilic counterpart, it may culminate in insolubility rather than aggregation driven by the minimization of interfacial free energy, ultimately yielding *NPs* instead of polymeric micelles. Conversely, when the hydrophilic component dominates, the copolymer may fail to generate aggregates. Evidently, the INU-PLA_1–4_ copolymers are likely to exist as a blend of *NPs* and polymeric micelles, corroborated by the quantification of the soluble fraction forming micelles. A reduction in copolymer solubility is discernible as the degree of DD_mol_% increases (e.g., comparing INU-PLA_1_ with INU-PLA_2_ and INU-PLA_3_ with INU-PLA_4_) along with variations in the length of the PLA (such as INU-PLA_1-2_ compared to INU-PLA_3–4_), as elucidated in Table 1.

### 3.2. Size, Shape, and Morphological Transitions

**Figure 2:**
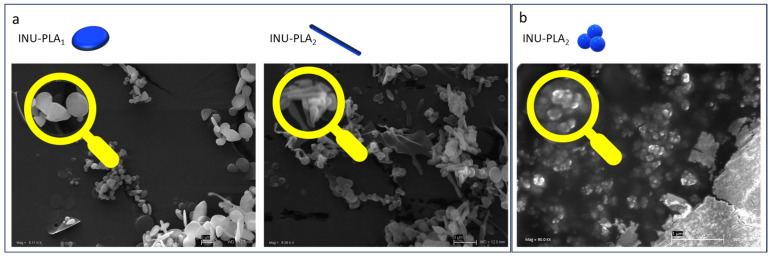
Disc, rod-like, and spherical INU-PLA aggregates: (**a**) samples obtained by pre-assembling/film rehydration method and (**b**) samples obtained via pre-assembling/dialysis method.

Numerous questions to be answered persist regarding the structure–property associations of graft copolymer micelles and *NPs*. It is important to highlight that information about systems of this nature has remained relatively limited compared to nanostructures founded on block copolymers so far in terms of investigation on certain key attributes: morphology, segment distribution within the core/shell/interface and phase transitions within these systems have been inadequately explored. In our prior investigation [20], we elucidated how variations in size and structure are contingent upon polymer hydrophilicity. Specifically, lower hydrophilicity leads to reduced interfacial curvature in the copolymer, thereby favoring the formation of vesicles over micelles/*NPs*. Those earlier findings also demonstrated that, depending on the chosen preparation method (whether nanoprecipitation or film rehydration), these systems exhibited the propensity to assemble into microparticles/micro-polymersomes or nanoaggregates. Through the exploration of novel conditions, we achieved a diverse array of both spherical and non-spherical morphologies via distinct preparation techniques applied to the same copolymer, i.e., INU-PLA_2_, as exemplified in Figure 2a,b.

Rod-like morphologies were achieved through the pre-assembling/film rehydration method. In this process, material films were obtained by freeze-drying dispersions (which contained assembled structures resulting from solvent displacement). These films were then rehydrated under bath sonication. When the method was changed to dialysis, nanospheres were obtained instead of rods from INU-PLA_2_. Likewise, while keeping the preparation method constant but modifying the copolymer composition, a noticeable morphological transition from the rod-like structure of INU-PLA_2_ to a disc-like configuration of INU-PLA_1_ was observed. It is important to note that the preparation of SEM samples involved directly casting the dispersions. It cannot be ruled out that irregular morphologies may have emerged during the solvent evaporation. Additionally, it should be mentioned that hydrophobically functionalized INU polymers had previously demonstrated the ability to form lipid bilayer nanodiscs [28].

### 3.3. Exposure of INU on the NP Surface

A FALT study was conducted to measure the thickness of the outer hydrophilic shell. The core concept of the experiment relies on utilizing the Gouy–Chapman theory as a foundational approximation. This experiment is conducted by observing how changes in ionic strength impact the surface ζ potential of particles [29].

The study showed how passing from INU-PLA_1,2_ to INU-PLA_3,4_ the thickness of the shell decreased. The worth of notice is that the thickness does not decrease gradually with the INU wt% but it goes from an average of 3.4 nm for INU-PLA_1_ and INU-PLA_2_ to 2.3 nm for INU-PLA_3_ and INU-PLA_4_. This leads to supposing that a discrete threshold condition exists, probably connected with the conformation of polymer molecules at the surface. At low coverage density, hydrophilic segments on the core surface shrink, taking a configuration referred to as a mushroom-like conformation. When two polymer chains approach each other because of the increasing coverage, they are stretched due to repulsive interaction (osmotic pressure and the compression of polymer chains between surfaces) and form a brush-like extended layer [30]. Thus, the strength of the steric repulsion between the corona chains, their conformation, and ultimately, the outer layer thickness are related to the chain density on the surface of the particle core.

To investigate the matter, the surface INU density (Γ) on *NPs* and the relative composition at the interface were determined by ^1^H NMR after the solvent displacement process in D_2_O. As expected, the higher the INU wt% in the *NPs*, the higher the INU surface density. A density below 95 INU/100 nm^2^ (found for INU-PLA_3–4_) corresponds to a shell thickness measured by FALT in the order of two times the radius of gyration (R_g_, Table 2), which is roughly the space occupied by the coil domain of a corona chain in a mushroom-like conformation [30].

Therefore, below a density of 95 INU/100 nm^2^, the hydrophilic segments of INU-PLA_3–4_ on the *NPs* surface assume a mushroom-like conformation. From 95 INU/100 nm^2^ and up to 220 INU/100 nm^2^ (found for INU-PLA_1–2_), the thickness of the hydrophilic shell increases discreetely, passing from 2.3 to 3.4 nm, indicating an extension of the shell forming chains. The 3.4 nm limit for INU-PLA_1-2_ seems somewhat limited compared to the extent of PEG elongation when used as a corona-forming polymer. This phenomenon might be clarified by understanding that INU shares similarities with a spiro-substituted PEG at its core, but the presence of fructosidic side chains results in a helical backbone structure for INU, contrasting with the extended worm-like structure typically seen in PEG [31]. Thus, the extension of the chain and the max shell thickness are somehow constrained by this factor. Moreover, being a non-block copolymer, hydrophobic residues can associate within the same polymer chain (i.e., intramolecularly) leading to the formation of loops [32] able of limited stretching.

A dimensionless parameter, σ, can be used to explore the density of the corona chains on the surface of the *NP* core in relation to various molecular parameters. σ is defined as the number of segments exposed on a unitary area of the NP times the square of the length of the repeating unit (a),

σ = (a^2^/Γ) × 100, (6)

when σ < a^2^/R_g_^2^, i.e., when the product σa^−2^R_g_^2^ < 1, each chain occupies a hemisphere of radius Rg (mushroom-like conformation) [33]. On the contrary, for σa^−2^R_g_^2^ > 1, chains have to adopt conformations that are extended in the direction perpendicular to the surface. As reported in Table 2, the σa^−2^R_g_^2^ product is found higher than unity for INU-PLA_1_ and INU-PLA_2_, being 2.68 and 1.15 on average, respectively. On the contrary, results for INU-PLA_3_ and INU-PLA_4_ show how INU segments on those particles’ surfaces are in a not overlapping condition falling in the low σ region.

Analogously with PEG, which can exhibit two main conformations, namely, mushroom and brush conformations [34], it is reasonable for INU to have the same behavior apparently. This opens a new scenario, and further investigation is required to find out the repercussions of conformation changes on NP stability and fate.

### 3.4. The Interplay between the Copolymer’s Hydrophilic and Hydrophobic Components

^1^H NMR measurement of INU-PLA_1–4_ in water after solvent displacement showed the signals of hydrophobic protons in D_2_O are suppressed but could still be observed. A possible explanation is represented by the generation of a diffused interface where solvophobic units and shell-forming segments form a swollen layer. This behavior that appears in crew-cut micelles formed by both block and gradient copolymers [35] seems to be common to our graft construct.

The detection of PLA residues by ^1^H NMR in D_2_O increases as the shell thickness increases. By comparing the integral of the signals of INU and PLA recorded both in D_2_O and DMSO-d6 (a suitable solvent for both the segments), INU and PLA exposure% were calculated. As can be seen from Table 3, a drastic increase (up to around 10%) in the detectable PLA exposed at the swollen interface was found for INU-PLA_1–2_ compared to INU-PLA_3–4_. This behavior is compatible with a scenario in which loops are formed on the surface: We could imagine that the stretching of the hydrophilic segments pulls out more solvophobic units towards the swollen interface.

This observation suggests that the density threshold found may correspond to a limit to the optimal balance between hydrophilicity and hydrophobicity that influences the structure and stability of these copolymer systems. Thus, INU-PLA_2_, with a thicker shell, diffused interface, small hydrodynamic diameter (<100 nm) after solvent displacement, and the lowest CAC value, was chosen as the best candidate for drug loading and in vitro biological testing.

### 3.5. Preparation and Characterization of INU-PLA2 NPs Loaded with ST

Hepatocellular carcinoma (HCC), the most prevalent form of liver cancer, poses a significant challenge due to its aggressive nature and alarmingly low rate of early-stage diagnosis, resulting in poor patient outcomes [36]. Consequently, it is imperative to improve the specificity and efficiency of treatment approaches for this debilitating condition. *ST* has emerged as a potent weapon against HCC cells and is globally recognized as a cornerstone of HCC first-line treatment. However, the clinical efficacy of *ST* is hindered by its inherent limitations, including low water solubility and high logP values [37]. The consequence of these unfavorable properties is the administration of *ST* necessitates multiple oral doses, often leading to severe toxicity and compromising its therapeutic usefulness. Thus, an increasing interest is in developing injectable nanomedicines that can address these limitations. Such nanosystems would offer the advantage of reducing the overall dose of *ST*, primarily through passive accumulation at the tumor site and sustained release, leading to improved therapeutic outcomes [38].

As discussed above, INU-PLA_2_ has been chosen to prepare *ST*-loaded *NPs* due to its optimal characteristics for stability prediction. *ST* was loaded into INU-PLA_2_ *NPs* using the pre-assembling/dialysis method to obtain *NPs* with spherical morphology. The incorporation of *ST* into INU-PLA_2_ *NPs* led to an increase in the solubility in water several times. A picture of the *NP* dispersion after the incorporation of *ST*, compared to *ST* dispersion treated the same way, is reported in Figure S5 (Supplementary Materials). A drug loading of 15% (EE > 90%) was achieved.

**Figure 3:**
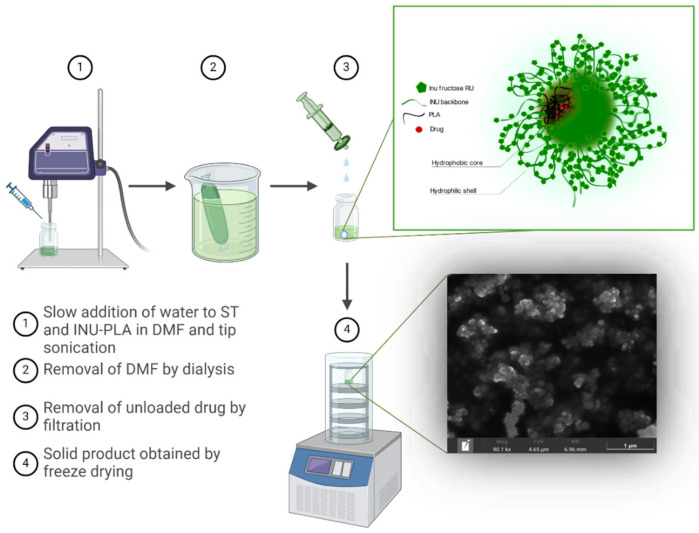
Schematic diagram of the preparation of *ST*-loaded *NPs* with a core–shell structure and a representative SEM image. Created with BioRender.com.

INU-PLA_2_/SF *NPs* showed a spherical shape, as can be seen by SEM micrographs (insert in Figure 3), confirming the presence of the loaded amount of drug within the NPs did not alter the assembly behavior and the morphology.

**Figure 4:**
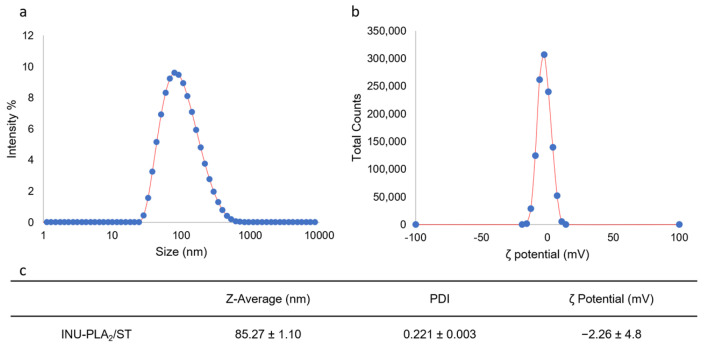
Dynamic and electrophoretic light scattering results for the determination of INU-PLA2/*ST* particle size and Zeta potential: (**a**) representative size distribution, (**b**) representative ζ potential distribution, and (**c**) mean values of Z-Average, PDI, and ζ potential (three independent measurements).

NM studies were performed to determine the average diameter, polydispersity index (PDI), and ζ potential of INU-PLA_2_/*ST NPs* in double distilled water (Figure 4). The *NPs*’ Z-Average is about 90 nm, with a low PDI of around 0.22, and the ζ potential was found to be slightly negative. Those data, obtained after redispersion of freeze-dried samples, testify to the colloidal stability of the system and the cryoprotectant ability conferred by INU on the particle surface.

The liberation of the drug from the INU-PLA_2_ nanoparticles was investigated in phosphate-buffered saline (PBS) with a pH of 7.4 through the dialysis method. This was carried out to assess the capacity of the synthesized nanoparticles to maintain the encapsulated drug. Because *ST* is poorly soluble in this medium, 10% (*v*/*v*) of Tween 80 was added to the release medium, as a diffusion-helping agent. After 24 h incubation, the amount of *ST* released from the produced *NP* sample was neglectable, while the free drug diffusion through the dialysis membrane reached around 75% *w*/*w* after the same time period (Figure S6, Supplementary Materials).

### 3.6. In Vitro Biological Characterization

**Figure 5:**
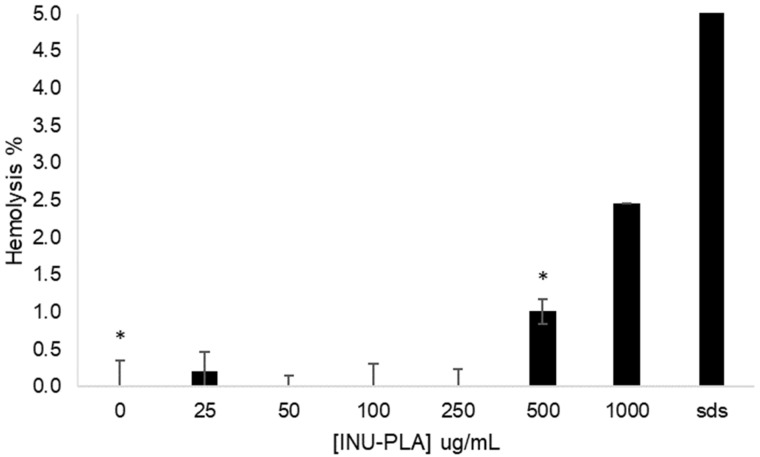
Analysis of hemolytic properties of INU-PLA_2_ *NPs* by spectrophotometric determination of the hemoglobin release from RBCs. * *p* ≤ 0.01.

In vitro hemocompatibility of INU-PLA_2_ was determined by hemoglobin release from RBCs after incubation with the copolymer for 1 h at 37 °C. Based on the guidelines outlined in the ASTM E2524-08 (2013) [39] standard, a percentage of hemolysis exceeding 5% typically signifies material-induced damage to red blood cells (RBCs). Nevertheless, none of the tested concentrations surpassed this threshold criterion. Even at the highest concentration of 1 mg/mL, the percentage of hemolysis slightly increased to an average of 2.46%, as shown in Figure 5. The effect on RBCs was also investigated by microscopy observation, to determine morphological changes and erythrocyte aggregation phenomena. Only at 1 mg/mL, a dysmorphic population of cells was observed (crenocytes), and in all the tested samples, erythrocyte aggregation was never observed. Representative micrographs of isolated RBCs after incubation with INU-PLA_2_ at 25, 500, and 1000 μg/mL are reported in Supplementary Materials (Figure S7). Overall, these findings suggest that INU-PLA_2_ holds the potential for intravenous administration without causing damage to RBCs.

Fluorescently labeled FITC-INU-PLA_2_ was synthesized using the same synthetic procedure to obtain INU-PLA, starting from commercially available INU-FITC instead of INU. Briefly, INU-FITC-PLA was obtained via one-pot alcoholysis by adding INU-FITC, previously treated with a base, to the imidazoline derivative of acid end-capped PLA. A final product with 0.006 mol% FITC (mol FITC/mol fructose repeating units × 100) equivalent to 14.55 × 10^−6^ mmol/mg % was obtained (DD mol% PLA = 6.63% mol PLA/mol fructose repeating units × 100; Mn NMR = 15,932 Da).

**Figure 6:**
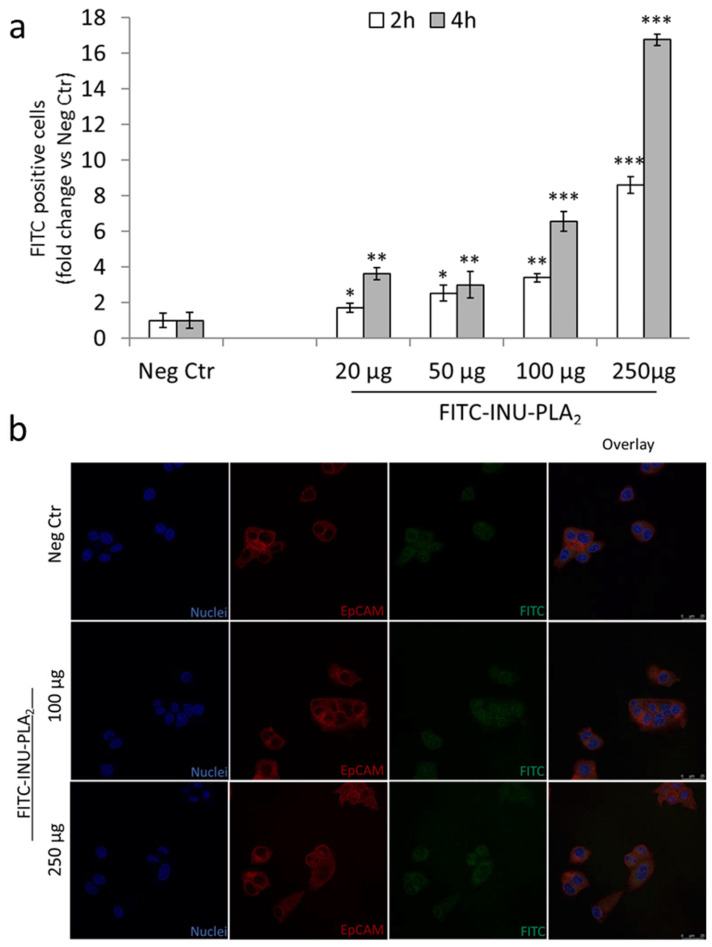
Cellular uptake studies on HepG2 cells. (**a**) FACS analysis of FITC-INU-PLA_2_ *NPs*’ internalization in HepG2 cells. Average values of FITC-positive HepG2 cells expressed as mean ± SD of three independent FACS experiments (* *p* < 0.05, ** *p* < 0.01, *** *p* < 0.005 vs. negative control). (**b**) Representative confocal microscopy images of FITC-INU-PLA_2_ (green fluorescence) uptake in HepG2 cells treated for 4 h with 100 µg or 250 µg of *NPs*. Nuclei were stained with DAPI (blue fluorescence), and EpCAM (red fluorescence) was used to stain the plasma membrane. Scale bar = 25 µm.

HepG2 cells were cultured and exposed to the fluorescent *NPs* at various concentrations ranging from 20 to 250 µg/mL. Cellular uptake was assessed using FACS and fluorescence microscopy. FACS analysis clearly shows that cellular uptake of FITC-INU-PLA_2_ *NPs* significantly occurs starting from the low concentration of 20 µg, in a dose- and time-dependent manner (Figure 6a), with a maximum effect at the highest concentrations, as confirmed also by confocal microscopy analysis (Figure 6b and Figure S8). The efficient cellular uptake of FITC-INU-PLA_2_ by HepG2 cells underscores the potential of these *NPs* as drug carriers for targeted therapy. Further investigations into the precise mechanisms of cellular entry are warranted to optimize drug delivery strategies.

**Figure 7:**
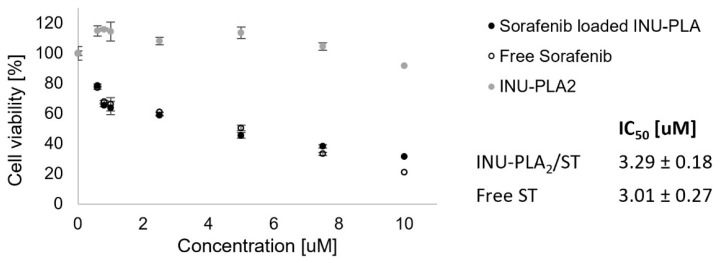
Antiproliferative effect and IC_50_ determination for free and *NPs* loaded *ST*.

The antiproliferative activity of free *ST* and INU-PLA_2_/*ST NPs* and the cytocompatibility of empty *NPs* were evaluated on HepG2 cells. The empty micelles showed no toxicity even after incubation for 24 h at the maximum NP concentration of 50 μg/mL. INU-PLA_2_/*ST NPs* and free *ST* showed the same IC_50_ after 24 h of incubation (3.29 ± 0.18 μM and 3.01 ± 0.27 μM, respectively) (Figure 7).

The observation that the IC_50_ of the loaded drug is comparable to that of the free drug is a significant finding. It implies that the encapsulation of *ST* within *NPs* does not compromise its antiproliferative activity against HepG2 cells. This suggests that the *NPs* may protect the drug from degradation or enhance its bioavailability, ultimately leading to a similar therapeutic effect. The apparent discrepancy of the antiproliferative effect with in vitro release studies reflects how these experiments, although crucial for initial evaluations, do not fully capture the complex interactions and dynamics at play within the cellular machinery of living organisms. Today, scientists actively research intracellular traffic and fate of nanoparticles and how these dynamics affect the efficacy of intracellular cargo release [40] and on the development and validation of a novel method for a predictive evaluation of drug liberation from the nanosystems [41].

## 4. Conclusions

This study focused on the structural characterization of micelles derived from graft INU-based amphiphiles. It aimed to uncover their unique features, drug-loading capabilities, and potential advantages for targeted drug delivery. The synthesized INU-PLA copolymers demonstrated intriguing hybrid properties and the ability to self-assemble into nanostructures, exhibiting excellent drug loading and sustained *ST* release. The structural properties of the micelles, including CAC, water solubility, carrier size, and morphology, were thoroughly investigated. Moreover, the in vitro biological characterization of HepG2 cells provided insights into the potential therapeutic benefits of these nanocarriers for HCC therapy. By elucidating the assembly behavior and functional properties of these INU-based micelles, this research contributes to the development of tailored nanomedicines for enhanced tumor treatment, offering valuable insights for the design and optimization of nanocarriers for targeted drug delivery in cancer therapy.

## Figures and Tables

**Table 1:** Molecular characteristics of INU-PLA_1–4_ copolymers in this study.

Sample	DD_mol_% PLA ^a^	Mn NMR (KDa)	INU wt%	CAC (×10^−3^M)	Solubility (mg/mL)
INU-PLA_1_	3.3 ^b^	12.3	54.5	8.5 × 10^−3^	>10
INU-PLA_2_	4.6 ^b^	13.7	46.2	4.9 × 10^−4^	>10
INU-PLA_3_	3.4 ^c^	16.2	36.9	3.6 × 10^−4^	0.94
INU-PLA_4_	7.6 ^c^	25.5	20.2	6.8 × 10^−4^	0.41

^a^ Degree of Derivatization expressed as moles PLA per 100 moles of INU fructose repeating units, calculated as reported in [20], by ^1^H NMR in DMSO. ^b^ Poly(D,L-lactide) acid endcap 1000–5000 Da was used in this derivative. ^c^ Poly(D,L-lactide) acid endcap 5000–10,000 Da was used in this derivative.

**Table 2:** NP characteristics as determined by DLS, ^1^H NMR, and FALT studies.

Sample	^a^ Z-Average (nm)	^a^ PDI	^b^ FALT (nm)	^c^ Γ	^d^ σ	^e^ σa^−2^R_g_^2^
INU-PLA_1_	167.4 ± 2.4	0.22 ± 0.03	3.4 ± 0.9	221.0 ± 15.9	0.218 ± 0.016	2.68 ± 0.19
INU-PLA_2_	98.6 ± 1.8	0.28 ± 0.01	3.4 ± 0.8	95.1 ± 0.3	0.094 ± 0.000	1.15 ± 0.00
INU-PLA_3_	85.3 ± 0.9	0.22 ± 0.00	2.2 ± 1.0	68.8 ± 3.7	0.068 ± 0.004	0.83 ± 0.05
INU-PLA_4_	93.6 ± 4.32	0.48 ± 0.01	2.4 ± 0.5	28.5 ± 3.3	0.028 ± 0.003	0.35 ± 0.04

^a^ Measured by DLS in water at 25 °C. ^b^ Measured by electrophoretic light scattering in water at 25 °C. ^c^ INU density on *NPs*’ core expressed as the number of INU molecules per 100 nm^2^, measured by ^1^H NMR after solvent displacement. ^d^ Calculated considering a ∼0.31 nm for INU. ^e^ R_g_ calculated accordingly with [31].

**Table 3:** INU and PLA exposure percentages as determined by ^1^H NMR in D_2_O and DMSO-d6 as a selective and a good solvent, respectively.

Sample	INU Exposure %	PLA Exposure %
INU-PLA_1_	97.2 ± 7.0	9.4 ± 1.15
INU-PLA_2_	86.2 ± 0.3	11.8 ± 0.4
INU-PLA_3_	88.8 ± 4.8	2.9 ± 0.3
INU-PLA_4_	60.5 ± 7.0	1.5 ± 0.1

## Data Availability

The raw data supporting the conclusions of this article will be made available by the authors upon request.

## Supplementary Materials

The following supporting information can be downloaded at https://www.mdpi.com/article/10.3390/pharmaceutics16080971/s1, Figure S1: The dispersity and the respective GPC chromatograms of poly(D,L-lactide) acid endcap 1000–5000 Da (a) and 5000–10,000 Da (b), as reported in Certificate of Analysis available at https://akinainc.com; Figure S2: Representative calibration curve obtained by measuring Abs at 270 nm of *ST* solutions in DMSO with known concentration in the range of 1 to 20 μg/mL; Figure S3: Plot of the ln of the zeta potential vs. 3.33 [NaCl]^0.5^ for the determination of Fixed Aqueous Layer Thickness (FALT) in INU-PLA_1-4_ nanoparticles; Figure S4: Quantification of INU on the nanoparticles surface by ^1^H NMR; Figure S5: Photographs of sorafenib in water and INU-PLA_2_/*ST* in water; Figure S6: *ST* diffusion after 24 h in PBS pH 7.4 Tween 80 1% *v*/*v*; Figure S7: Micrographics of isolated RBCs after incubation with INU-PLA_2_ at the concentration of 25, 500, and 1000 μg/mL. For comparison, positive (SDS) and negative (DPBS) controls are reported.
