# Supplementary material for: Inulin Amphiphilic Copolymer-Based Drug Delivery: Unraveling the Structural Features of Graft Constructs
Source: Pharmaceutics. 2024 Jul 23;16(8):971. doi: 10.3390/pharmaceutics16080971 (PMC11359108; doi:10.3390/pharmaceutics16080971)
Supplement: Supplementary file 1 [file pharmaceutics-16-00971-s001.zip › pharmaceutics-3065404-supplementary.pdf]

Supplementary Materials

a

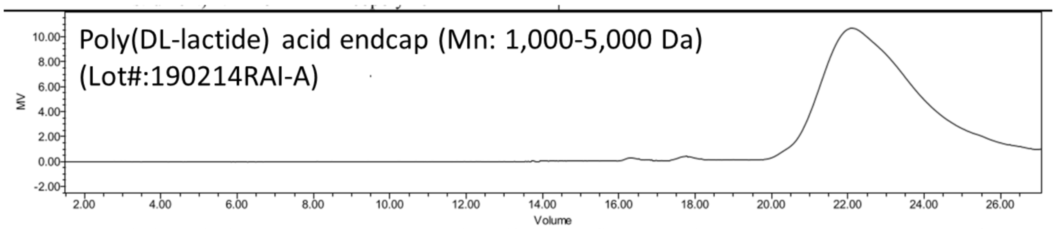

GPC Analysis Method: Waters Breeze 2 system with 1 ml/min THF flow across three GPC columns. Detection via refractive index, calibrated against polystyrene standards.

Polymer	M_n (from GPC)	M_w (from GPC)	PDI
PDLLA	3,777	5,295	1.40

b

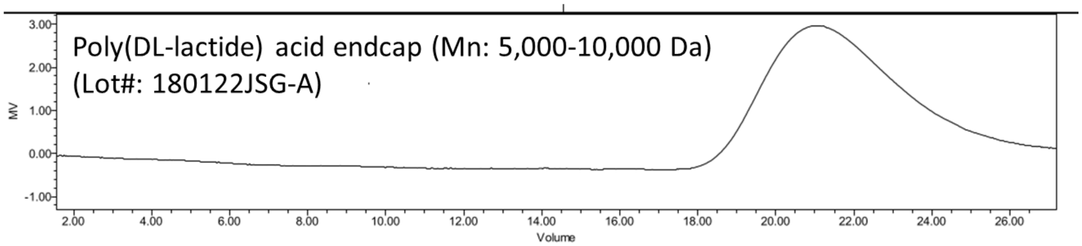

GPC Analysis Method: Waters Breeze 2 system with 1 ml/min THF flow across three GPC columns. Detection via refractive index, calibrated against polystyrene standards.

Polymer	M_n (from GPC)	M_w (from GPC)	PDI
P(DL)LA	7,691	13,057	1.69

Figure S1. The dispersity and the respective GPC chromatograms of Poly(D,L-lactide) acid endcap 1000-5000 Da (a) and 5000-10000 Da (b), as reported in Certificate of Analysis available at https://akinainc.com/polyscitech/products/polyvivo/Table-PEG-PLA-PLGA-PCL-copolymers-and-linear-water-soluble-fluorescent-intermediate-polymers.php

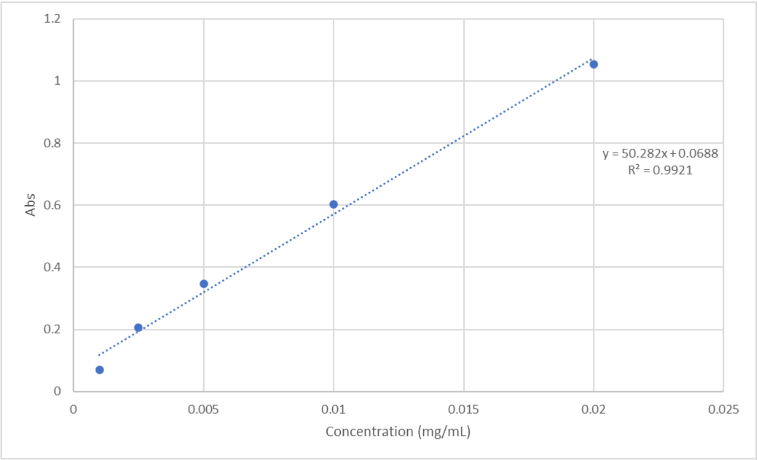

Figure 2. Representative calibration curve obtained by measuring Abs at 270 nm of ST solutions in DMSO with known concentration in the range of 1 to 20 µg/mL.

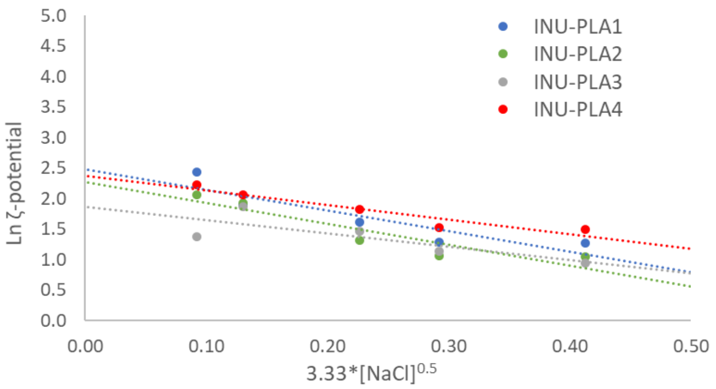

Figure S3. Plot of the ln of the zeta potential vs 3.33 [NaCl]$^{0.5}$ for the determination of Fixed Aqueous Layer Thickness (FALT) in INU-PLA$_{1-4}$ nanoparticles.

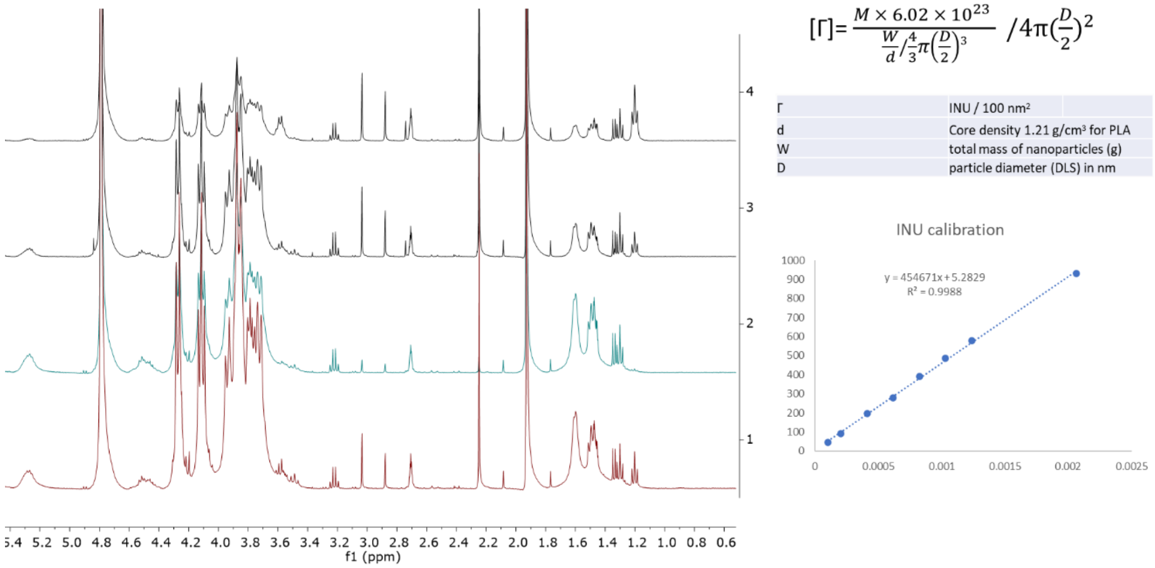

Figure S4. Quantification of INU on the nanoparticles surface by ^{1}H NMR.

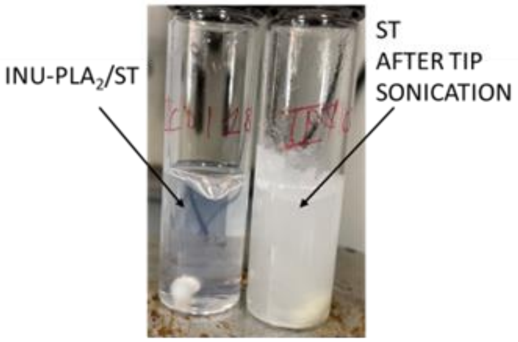

Figure S5. Photographs of sorafenib in water and INU-PLA$_2$/ST in water

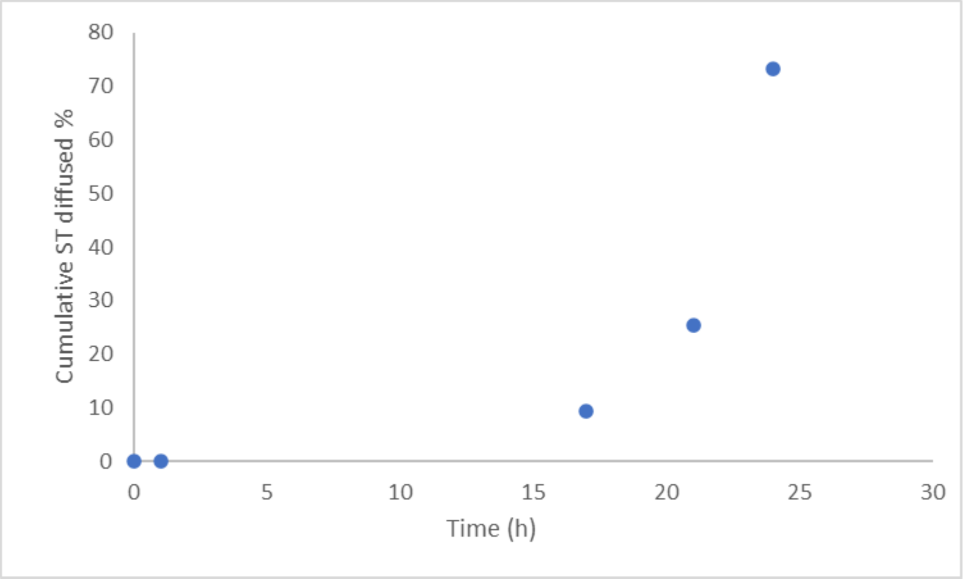

Figure S6. ST diffusion after 24h in PBS pH 7.4 Tween 80 1% v/v.

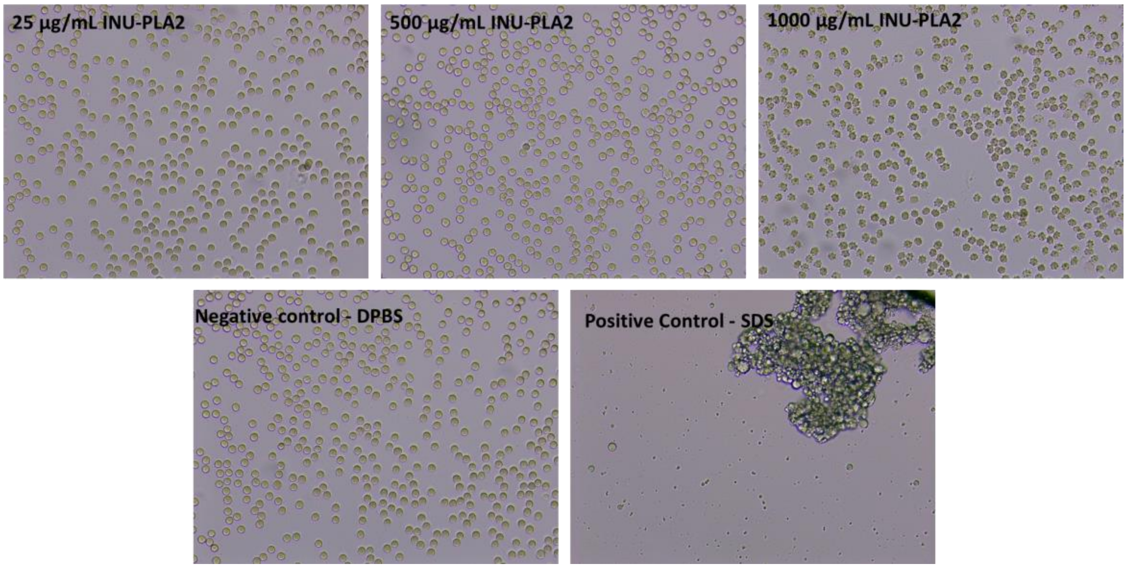

Figure S7. Micrographics of isolated RBCs after incubation with INU-PLA2 at the concentration of 25, 500 and 1000 µg/mL. For comparison, positive (SDS) and negative (DPBS) controls are reported.

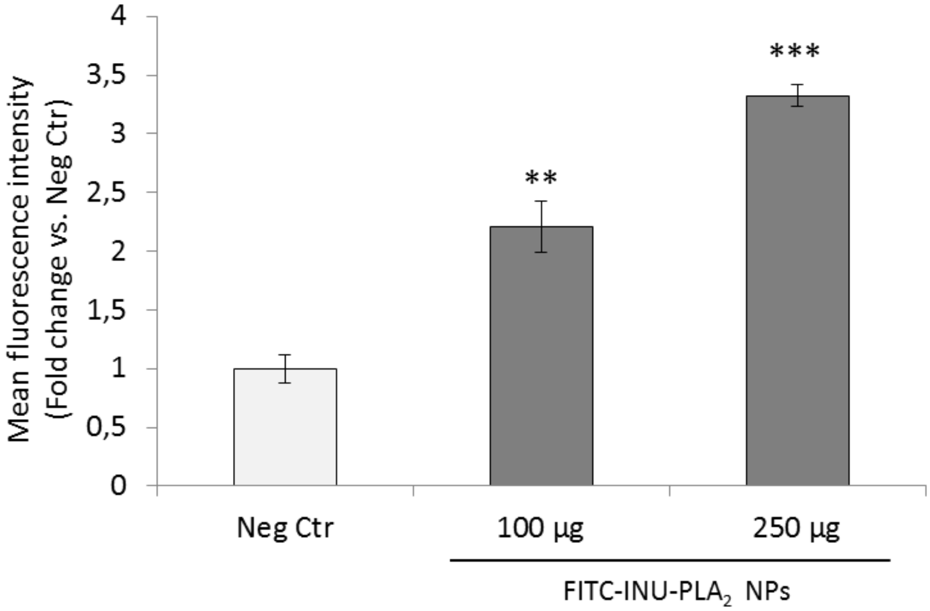

Figure S8. Fluorescence intensity for FITC-INU-PLA2 NPs signals in HepG2 cells. The FITC fluorescence signal was quantified using ImageJ software by analyzing three randomly selected microscopic fields for each coverslip slide. A total of 9 microscopic fields (3 x 3 coverslip slide independently prepared) were analyzed for each treatment.. Data are expressed as Mean ± SD **p < 0.01, ***p < 0.005 vs. Negative control
